# Predictors of In-Patient Mortality of Severe Acute Malnutrition of Hospitalised Children in a Tertiary Facility in Southern Nigeria

**DOI:** 10.7759/cureus.24195

**Published:** 2022-04-16

**Authors:** Joanah M Ikobah, Kelechi Uhegbu, Francis Akpan, Leo Muoneke, Emmanuel Ekanem

**Affiliations:** 1 Paediatric Gastroenterology, Hepatology and Nutrition Division, Department of Paediatrics, University of Calabar, University of Calabar Teaching Hospital, Calabar, NGA; 2 Paediatric Gastroenterology, Hepatology and Nutrition Division, Department of Paediatrics, University of Calabar Teaching Hospital, Calabar, NGA; 3 Department of Paediatrics, University of Calabar Teaching Hospital, Calabar, NGA

**Keywords:** nigeria, mortality, predictors, children, inpatient, sam, severe acute malnutrition

## Abstract

Introduction

Severe acute malnutrition (SAM) remains a public health concern in developing countries. Children with SAM are nine times more likely to die compared with those that are well-nourished. Most studies on SAM in Nigeria focus on disease burden without evaluating risk factors that may be useful as interventions in reducing mortality. This study evaluated predictors of mortality and outcome among hospitalised children with SAM in Southern Nigeria.

Methods

Children with SAM admitted into the paediatric medical ward of the University of Calabar Teaching Hospital between September 2017 and November 2019 were studied prospectively. A multivariable logistic regression was used to identify factors that independently predicted mortality with a p-value <0.05 considered significant.

Results

One hundred children were studied. The mean age was 14.28 ± 14.04 months, of which 89% were less than two years of age. Oedematous and non-oedematous SAM were found in 18.5% and 81.5%, respectively. Co-morbidities included tuberculosis (TB) (13.0%), HIV (12.0%), and HIV/TB co-infection (3.0%). Clinical presentation included fever (21.7%), anaemia (19.9%), diarrhoea (19.1%), skin changes (8.7%), and shock (1.8%). The mean duration of hospital stay was 11.48 ± 6.87 days. Nine of the children were discharged against medical advice and were excluded from further analysis. About 92.3% were discharged for follow-up and 7.7% died. After multivariable regression, the predictors of mortality were shock (p=0.037, adjusted odds ratio (aOR): 17.51, 95% confidence interval (95% CI): 1.19-258.77) and skin changes (p=0.035, aOR: 9.81, 95% CI: 1.18-81.46).

Conclusion

The presence of shock and skin changes are independently associated with mortality in hospitalised children with SAM. Prompt referral of children with SAM and more so with complications of shock and skin changes is hereby advocated to reduce mortality.

## Introduction

Globally, about 16 million children under five years of age suffer from severe acute malnutrition (SAM), with two-thirds of these children living in South-East Asia and one-third in Africa [1,2]. Undernutrition is estimated to contribute to about 45% of childhood mortality [2]. Children with SAM are nine times more likely to die compared to healthy children [2]. World Health Organisation (WHO) classifies SAM into uncomplicated and complicated SAM based on the presence or absence of medical complications such as the presence of infections, oedema, and lack of appetite [1].

Although there is a standard protocol for the management of SAM in children, case-fatality rates in hospitals remain unacceptably high at between 30% and 50%, especially in children with co-morbidities and medical complications including infections, diarrhoea, shock, and anaemia [1]. In Southeast Nigeria, the mortality rate in a tertiary hospital for children with protein-energy malnutrition was 40.1% [3]. A community house-to-house survey carried over two decades ago in Cross River State, Nigeria among 471 total deaths over a one-year period of children under five years of age showed protein-energy malnutrition accounted for 11% of the mortality [4].

The Nigerian Demographic and Health Survey (NDHS) of 2018 showed that 37% of children under five years of age are stunted and 7% are wasted [5]. Most studies on SAM in Nigeria focussed on disease burden without evaluating risk factors, which may be useful in reducing mortality in hospitalised children with SAM. This study assessed the predictors of mortality and duration of hospital stay of children with SAM admitted into the paediatric medical ward of a tertiary hospital in Southern Nigeria.

## Materials and methods

Study setting

The study was conducted in the Department of Paediatrics at the University of Calabar Teaching Hospital (UCTH), Calabar, Cross River State, South-South Nigeria. Cross River State has a population of 3.86 million people [6]. Calabar is the capital city of the state, with its inhabitants mainly Efiks, Ibibios, Ejaghams, and other ethnic groups. Occupation of the dwellers commonly includes civil service, farming, fishing, trading, artisans, and other works of life. This is the only tertiary hospital in the state and serves as a referral centre for general hospitals and private hospitals across the state and beyond. Patients were admitted into the paediatric nutrition unit, which is in the paediatric medical ward, and were managed by medical personnel trained in paediatric nutrition. The WHO protocol for the management of SAM in children was used for treatment.

Study design

This was a two-year prospective cohort quantitative study of children admitted with severe acute malnutrition.

Study participants

Children admitted for SAM between September 2017 and November 2019 whose parents or caregivers gave informed consent were recruited into the study. A total of 100 children who presented with severe acute malnutrition during the study period were enrolled in the study. Children with SAM who are also diagnosed with other medical conditions such as sickle cell disease, malignancies, congenital malformations, chronic liver disease, chronic kidney disease, and neurological impairments were excluded from the study.

Data collection

Information on socioeconomic and demographic factors of patient and family, feeding practice-related variables including breastfeeding history, time of introduction of solid, semi-solid, or soft foods, types of foods given, clinical symptoms patient presented with were obtained from caregivers using a semi-structured questionnaire. Patients' hospital notes were also reviewed, and relevant data were extracted on medical complications, co-morbidities, duration of hospital stay, type of malnutrition, and treatment outcome. The WHO criteria for the management of SAM were used in this study as criteria for admission, diagnosis, and discharge. The outcome measured was mortality during admission. Family socioeconomic status determination was based on Ogunsanya’s classification [7].

Diagnostic criteria for severe acute malnutrition

The WHO diagnostic criteria for SAM weight for length/height (WFH) less than −3 z-score, mid-upper arm circumference (MUAC) less than 11.5 cm and/or the presence of oedema were used [8]. Children without oedema were classified as having non-oedematous SAM and those with oedema as having oedematous SAM [8]. Weight was measured using an infant Waymaster weighing scale and stadiometer (health scale) depending on the child’s age.

Admission criteria for severe acute malnutrition 

Admission criteria for children with SAM were based on the WHO criteria. Children with weight for length/height <−3 z-score, mid-upper arm circumference (MUAC) <11.5 cm, presence of bilateral oedema, presence of medical complications, and children who failed the appetite test, that is, failed to eat a pre-specified amount of food relative to their weight [9]. Children with SAM aged less than six months were also admitted.

Discharge criteria

This was based on the WHO criteria for discharge of children with SAM [8].

Ethical consideration

Ethical clearance for the conduct of this study was obtained from the University of Calabar Teaching Hospital, Health Research Ethics Committee (UCTH/HREC/33/714). Informed consent was obtained from mothers of participating children verbally after addressing each mother about the study.

Statistical analysis 

Data were analysed using Statistical Package for Social Sciences (SPSS) for Windows, Software Version 22.1. (SPSS Inc., Chicago, IL, USA). Variables that follow a normal distribution were described with means and standard deviations. The Chi-square and Fisher’s exact tests were used to test for differences in proportion or mean between groups. Multivariable logistic regression was used to identify factors that independently predicted mortality and odds ratios (OR) with a 95% confidence interval (CI) were reported. A p-value <0.05 was considered significant.

## Results

Sociodemographic characteristics

A total of 100 children were admitted for SAM during the study period. The mean age was 14.28 ± 14.04 months with a median of 11.0 months and interquartile range of 6-17 months. Most of the children (65.0%) were infants aged 1.5 to 12 months of age, followed by children aged 13-24 months (24.0%). There were more females (54.0%) than males (46.0%). None of the children came from a high social class family background but from the low (88.0%) and middle (12.0%) social class. Mean maternal and paternal ages were 28.09 ± 6.39 and 35.60 ± 8.6 years, respectively. This is shown in Table 1.

**Table 1:** Sociodemographic characteristics of children with SAM (n = 100). SAM: severe acute malnutrition.

Variables	Mean ± SD	Frequency	Percentage (%)
Age group (months)			
1–12	14.28 ± 14.04	65	65.0
13–24		24	24.0
25–36		4	4.0
37–48		2	2.0
49–60		5	5.0
Sex			
Male		46	46.0
Female		54	54.0
Social class			
High		0	0.0
Middle		12	12.0
Low		88	88.0
Parent’s marital status			
Married living together		69	69.0
Married not living together		2	2.0
Co-habiting		14	14.0
Single parenting		15	15.0
Number of siblings			
0–3		62	62.0
>3		38	38.0

Antenatal and nutritional history of children admitted for SAM

As shown in Table 2, 76 (76.0%) mothers attended antenatal care (ANC) and 36.3% delivered in non-tertiary hospitals, 24.2% had home delivery, 19.8% delivered in tertiary hospitals, 17.6% were at the traditional birth attendant (TBA) home, and 2.2% took place in the church. Exclusive breastfeeding (EBF) for six months was noted in 15.1% of infants. About 40.9% breastfed for less than six months, 26.9% practiced mixed feeding, and 17.2% did not breastfeed. Most children (61.0%) were fed with homemade complementary food. The consistency of the feeds was reported to be watery by 55.0% of the caregivers. A majority of the caregivers (72.7%) did not add a protein source to the complementary food.

**Table 2:** Antenatal and nutritional characteristics of children with SAM (n = 100). SAM: severe acute malnutrition.

Variables	Frequency	Percentage (%)
Antenatal care		
Yes	76	76.0
No	24	24.0
Place of delivery		
Tertiary hospitals	18	18.0
Other hospitals	33	33.0
Traditional birth attendant	16	16.0
Home delivery	22	22.0
Church delivery	2	2.0
Missing	9	9.0
Breastfeeding history		
Exclusively breastfed for six months	14	14.0
Breastfed for less than six months	38	38.0
Not breastfed at all	16	16.0
Mixed feeding	25	25.0
Type of complementary food given		
Commercial	9	9.0
Homemade	61	61.0
Mixed	30	30.0
Consistency of feed		
Watery	55	55.0
Thick	44	44.0
Who feeds the child?		
Mother	69	69.0
Family members	5	5.0
Nanny	1	1.0
Mixed	3	3.0

Type of SAM and clinical characteristics of admitted children

**Figure 1:**
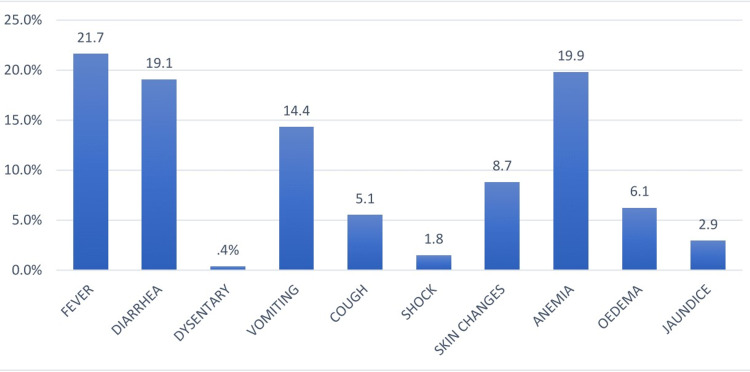
Clinical presentation of children admitted for SAM. SAM: severe acute malnutrition.

Oedematous SAM occurred in 18.5% of the children, and 81.5% had non-oedematous SAM. Co-morbidity included tuberculosis (13.0%), HIV/HIV exposed status (12.0%), and HIV/TB co-infection (3.0%), respectively. Clinical presentation among the admitted children was fever (21.7%), anaemia (19.9%), diarrhoea (19.1%), skin changes (8.7%), oedema (6.1%), and shock (1.8%), as shown in Figure 1.

Outcome of management of children admitted for SAM

The mean duration of hospital stay on admission was 11.48 ± 6.87 days, with a range of 1 to 35 days. Nine of the children were discharged against medical advice (DAMA). Out of the 91 children left, 92.3% survived and were discharged for follow-up in the outpatient clinic, and 7.7% died.

Predictors of mortality among children admitted for SAM

Table 3 and Table 4 show significant associations were found between the presence of HIV infection (p=0.036), presence of skin changes (p=0.044), anaemia (p=0.020), and shock (p=0.048) and mortality among children admitted for SAM. However, age group, sex, social class of caregivers, breastfeeding history, type of SAM, presence of tuberculosis, fever, diarrhoea, and vomiting were not significantly associated with mortality. 

**Table 3:** Relationship between sociodemographic, nutritional, and clinical factors management outcome among children admitted for SAM. SAM: severe acute malnutrition, TB: tuberculosis. *Significant p-value and **Fisher’s exact test.

Sociodemographic factors	Management outcome		Chi-square value (X^2^)	p-value
	Survived (%)	Died (%)		
Age group (months)				
1-23	74 (93.7)	5 (6.3)	1.57	0.230**
≥24	10 (83.3)	2 (16.7)		
Sex				
Male	37 (88.1)	5 (11.9)	1.95	0.242**
Female	47 (95.9)	2 (4.1)		
Social class				
Middle	11 (100.0)	0 (0.0)	1.04	0.592**
Low	73 (91.2)	7 (8.8)		
HIV infection				
Yes	76 (95.0)	5 (5.0)	6.76	0.036*
No	8 (72.7)	3 (27.3)		
Tuberculosis				
Yes	72 (91.1)	7 (8.9)	1.15	0.588**
No	12 (100.0)	0 (0.0)		
TB/HIV co-infection				
Yes	83 (93.3)	6 (7.7)	5.16	0.149**
No	1 (50.0)	1 (50.0)		
Fever				
Yes	48 (88.9)	6 (11.1)	1.90	0.241**
No	33 (97.1)	1 (2.9)		
Diarrhoea				
Yes	42 (89.4)	5 (10.6)	0.93	0.445**
No	38 (95.0)	2 (5.0)		
Vomiting				
Yes	29 (85.3)	5 (14.7)	3.35	0.105**
No	51 (96.2)	2 (3.8)		

**Table 4:** Relationship between sociodemographic, nutritional, and clinical factors management outcome among children admitted for SAM. SAM: severe acute malnutrition. *Significant p-value and **Fisher's exact test.

Sociodemographic factors	Management outcome		Chi-square value (X^2^)	p-value
	Survived (%)	Died (%)		
Skin changes				
Present	16 (80.0)	4 (20.0)	5.13	0.044*
Absent	65 (95.6)	3 (4.4)		
Anaemia				
Present	44 (86.3)	7 (13.7)	5.52	0.020*
Absent	37 (100.0)	0 (0.0)		
Shock				
Present	3 (60.0)	2 (40.0)	7.55	0.048*
Absent	79 (94.0.0)	5 (6.0)		
Type of SAM				
Oedematous	14 (93.3)	1 (6.7)	0.07	1.000**
Non-oedematous	63 (91.3)	6 (8.7)		
Breastfeeding history				
Not breastfed at all	13 (92.9)	1 (7.1)	0.03	1.000**
Breastfed	64 (91.4)	6 (8.6)		

As shown in Table 5, only two of the independent variables (presence of skin changes and shock) made unique contributions to the model out of the five independent variables. It was shown that the odds of dying following management for SAM among children who presented with skin changes were 9.8 times the odds of dying among children who did not present with skin changes, while the odds of dying among those that presented with shock were 17.5 times the odds of those that did not.

**Table 5:** Multivariable analysis of predictors of management outcome of SAM. aOR: adjusted odds ratio, SAM: severe acute malnutrition, and TB: tuberculosis.

Predictors	Number	P-value	aOR	95% CI
HIV infection				
Infection	11		1	
No infection	77	0.053	0.11	0.01–1.02
TB/HIV co-infection				
Co-infection	2		1	
No co-infection	86	0.121	0.06	0.00–2.12
Presence of skin infection				
No	68		1	
Yes	20	0.035*	9.81	1.18–81.46
Presence of anaemia				
No	51		1	
Yes	37	0.998		
Presence of shock				
No	83		1	
Yes	5	0.037*	17.51	1.19–258.77

## Discussion

The number of children hospitalised with severe acute malnutrition continues to rise in Sub-Saharan Africa. Over the study period, 8.4% of the total children admitted had SAM. In this study, there was no significant difference in the gender of the children with SAM. This is in keeping with the study by Ubesie et al. [3] in Enugu and Cartmell et al. [9] in Maputo. A total of 79% of the study population were less than two years of age. Studies have shown that undernutrition is more common within this age bracket [3,10]. This emphasises the importance of the first 1000 days of life where exposure to poor nutrition increases the odds of stunting, morbidity, and mortality [11].

Children from the low socio-economic class accounted for 88% of the study population. This conforms to SAM's being a nutritional disorder of poverty and ignorance as underlining causes of SAM [12]. Alongside poverty is the illiteracy of mothers. Low socioeconomic status has been shown as both a basic cause of SAM at the national or regional level and an immediate cause at individual levels according to the conceptual framework of the determinants of undernutrition by the United Nations Children's Fund (UNICEF) [12]. This adversely affects the ability of families to purchase adequate, nutritious foods. Nahar et al. found that children severely underweight are more likely to have undernourished, poorly educated young mothers and poorly educated, unskilled fathers [13].

Exclusive breastfeeding and the introduction of solids, semi-solid, or soft foods at the appropriate time are part of the population-based indicators for assessing infant and young child feeding (IYCF) practices established by WHO [12]. IYCF contributes significantly to reducing childhood undernutrition when practiced effectively [12]. In this study, 16% of mothers never breastfed, making use of breastmilk substitute (BMS), which could lead to diarrhoea and undernutrition, especially when inappropriately prepared. Exclusive breastfeeding (EBF) is seen as the single largest preventive intervention outcome against childhood mortality, with a 13% reduction in under-five mortality compared to other interventions [14]. Optimal breastfeeding alongside complementary feeding could prevent malnutrition and save about a million children's lives [15]. Breastfeeding has been shown to reduce mortality in infants from diseases such as diarrhoea and provides immunoglobulins that aid in faster recovery during illness [15]. In this study, diarrhoea accounted for 19.1% of the children who presented with medical complications. Diarrhoea in the setting of inadequate nutritional intake could lead to SAM, and SAM could give rise to diarrhoea following an increased risk of infection or due to carbohydrate intolerance [8]. In addition, Meremiku et al. showed that failure to breastfeed was associated with 36.4% of underweight and 1.2% of persistent diarrhoea in a case-control study of children aged less than three years in a diarrhoea treatment unit in Calabar, Nigeria [16]. Most of the children in this study were given homemade complementary feeds (61%) as against commercially prepared complementary foods. About 72.5% had no protein in any form added to their feeds. Most homemade feeds given in this locality consist of cereal gruel, which is given in a light consistency (55.2%). Common cereal gruels given include maize, guinea corn, and wheat. Though maize is a staple food in Nigeria and a good source of nutraceuticals, its mode of preparation could lead to the loss of 20-25% of its nutrients [17]. It has poor protein quality and is low in levels of lysine and tryptophan [17]. 

Unfortified homemade complementary food contributes to micronutrient deficiency, which is a major contributor to childhood morbidity and mortality. A comparative study in Kano of children with SAM and healthy controls on the assessment of micronutrient deficiency showed micronutrient deficiency in both groups but worse in children with SAM [18]. In developing countries, possibly due to poor water supply and inappropriate food preparation methods, it may be difficult to prepare and store complementary food at home free of microbial contamination. Islam et al. [19] working in villages and urban slums of Bangladesh investigated the microbial quality of complementary foods and their association with diarrhoeal morbidity and nutritional status and showed that 40% of the complementary food samples were contaminated with *Escherichia coli*. Consumption of contaminated food was associated with a higher frequency of diarrhoea and malnutrition in the study population. Where home-prepared complementary foods are hygienically feasible, there may be a need to top up the diets of these children with adequate protein, energy, and fat. 

About 81.5% of the patients had non-oedematous SAM. This is in keeping with most studies in Africa that have shown non-oedematous SAM as the commonest type [3,10,19,20]. However, studies in Ethiopia [21] and South Africa [22] showed oedematous SAM as the commonest type. This may be due to different causes of undernutrition in different localities and could also be linked to the common food types used as weaning foods in different countries.

The presence or absence of oedema was not independently associated with mortality in this study. Karunaratne et al. [23] in a systematic review and meta-analysis on predictors of mortality in hospitalised children with SAM did not show an association between oedema and mortality.

The mean duration of stay on admission in this study was 11.48 ± 6.87 days. This was within the minimum international standard set for the management of SAM, with an average length of stay of fewer than 30 days [24]. However, the overall mean duration of stay on admission was shorter than in other studies [3,20]. This could be due to the underlying medical conditions of children in our study population and possibly the level of expertise in managing the patients. The mortality rate in this study was 7.7%. This was within the minimum international standard for in-patient management of severe acute malnutrition of less than 10% [24]. It was also lower than in other studies in Nigeria [3,25], Malawi [26], Sudan [20], and Ethiopia [27]. The low rate may be attributed to starting our patients on 50 kcal/kg/day of feeds in place of the recommended F75 by WHO, thereby reducing the risk of refeeding syndrome.

On univariate analysis, the presence of HIV infection, anaemia, skin changes, and shock were significantly associated with mortality in children with SAM. Following multivariable regression, children with skin changes and shock had a significant independent association with mortality. Those with shock had 17.5 times higher odds of death compared to those without shock. Wagnew et al. [27] in Ethiopia showed that children with shock were more likely to die compared to those without these medical complications. This finding was also upheld by Gebremichael et al. [28] and Guesh et al. [29]. Skin changes such as hypopigmentation, hyperpigmentation lesions, bullae formation, and desquamations were noted amongst the study population. In SAM, skin involvement varies, involving severe forms such as lichenoid skin changes, which have been shown to have a significantly poor outcome [30]. The strength of this study included the prospective design where data were collected at admission without bias to the outcome. One limitation of our study was that, as a tertiary hospital-based study, the findings may not completely reflect the situation at the lower tiers of the health system or in the communities.

## Conclusions

The overall mortality rate was 7.7%, with a mean duration of hospital stay of 11.48 days. The mortality rate was lower than the WHO estimated case fatality rate for in-patients with SAM. The presence of shock and skin changes has been demonstrated to be independently associated with mortality in children with SAM. Evidence-based guidelines for the treatment of shock in children with SAM are urgently needed.
